# Crystal structure and metal binding properties of the periplasmic iron component EfeM from *Pseudomonas syringae* EfeUOB/M iron-transport system

**DOI:** 10.1007/s10534-022-00389-2

**Published:** 2022-03-29

**Authors:** Mohan B. Rajasekaran, Rohanah Hussain, Giuliano Siligardi, Simon C. Andrews, Kimberly A. Watson

**Affiliations:** 1 School of Biological Sciences, Health and Life Sciences Building, University of Reading, Whiteknights Campus, Reading, RG6 6EX UK; 2 B23 Beamline, Diamond Light Source, Harwell Science Innovation Campus, Chilton, Didcot, OX11 0DE UK; 3 Present Address: Sussex Drug Discovery Centre, School of Life Sciences, University of Sussex, Falmer, Brighton, BN19QJ UK

**Keywords:** X-ray crystallography, EfeM, Peptidase-M75 domain, Acidic patch, Metal-binding, EfeUOB iron-transport system

## Abstract

EfeUOB/M has been characterised in *Pseudomonas syringae* pathovar. *syringae* as a novel type of ferrous-iron transporter, consisting of an inner-membrane protein (EfeU_Psy_) and three periplasmic proteins (EfeO_Psy_, EfeM_Psy_ and EfeB_Psy_). The role of an iron permease and peroxidase function has been identified for the EfeU and EfeB proteins, respectively, but the role of EfeO/M remains unclear. EfeM_Psy_ is an ‘M75-only’ EfeO-like protein with a C-terminal peptidase-M75 domain (EfeO_II_/EfeM family). Herein, we report the 1.6 Å resolution crystal structure of EfeM_Psy_, the first structural report for an EfeM component of *P. syringae* pv*. syringae*. The structure possesses the bi-lobate architecture found in other bacterial periplasmic substrate/solute binding proteins. Metal binding studies, using SRCD and ICP-OES, reveal a preference of EfeM_Psy_ for copper, iron and zinc. This work provides detailed knowledge of the structural scaffold, the metal site geometry, and the divalent metal binding potential of EfeM. This work provides crucial underpinning for a more detailed understanding of the role of EfeM/EfeO proteins and the peptidase-M75 domains in EfeUOB/M iron uptake systems in bacteria.

**Supplementary Information:**

The online version contains supplementary material available at 10.1007/s10534-022-00389-2.

## Introduction

Iron is an essential nutrient for all living organisms since it is required as a cofactor in a wide range of crucial biological processes. The safe handling and acquisition of sufficient quantities of iron are a major challenge for the vast majority of living organisms, especially bacteria. To overcome such difficulties, bacteria have developed sophisticated iron homeostatic mechanisms, which involve integration of four key processes: iron uptake, iron rationing, storage of excess iron and tight regulatory control (Andrews 1998; Andrews et al. 2003; Frawley and Fang 2014).

Among these processes, uptake of iron plays a crucial role in combating iron restriction for bacteria. This is particularly the case for bacterial pathogens that must overcome the host’s iron-withdrawing nutritional immunity defenses in order to mount a successful infection (Andrews et al. 2003). Several types of iron transporters are known in bacteria, which tend to be specific for either ferrous or ferric iron (Andrews et al. 2003; Caza and Kronstad 2013). The most common type of transporters are the ferric-specific siderophore systems, which are dependent on elaboration and secretion of low molecular weight ferric-chelating compounds such as siderophores (Andrews et al. 2003; Chu et al. 2010; Wandersman and Delepelaire 2004). Under anaerobic conditions, the reduced ferrous form of iron predominates, and many bacteria deploy anaerobic (or microaerobic) iron-uptake systems (Andrews et al. 2003; Hantke 1987). The FeoAB iron uptake system is such an example, which is ferrous-iron specific and is induced by anaerobiosis and iron restriction (Cartron et al. 2006; Hantke 1987, 2003; Kammler et al. 1993; Lau et al. 2016). Bacteria also obtain iron from various alternative sources. For example, transferrin, lactoferrin, haem, haemoglobin (representing 65% of human iron content) and haemopexin are just some of the other important direct iron sources (Andrews et al. 2003; Braun et al. 1998; Caza and Kronstad 2013; Genco and Dixon 2001; Sandy and Butler 2009; Wandersman and Delepelaire 2004).

Previously, we characterized in detail the *efeUOB* encoded EfeUOB system (previously designated YcdNOB), as a novel tripartite ferrous-iron transporter stimulated by low pH and activated both aerobically and anaerobically (Cao et al. 2007). This novel ferrous iron transporter is partially related to the Ftr1p-Fet3p mediated ferrous iron uptake in *Saccharomyces cerevisiae*, which comes under reductase-dependent, high-affinity Fe^2+^ transport (Askwith and Kaplan 1997; De Freitas et al. 2003; Taylor et al. 2005). Ftr1p is a multi-pass membrane protein (~ 45 kDa) with seven transmembrane domains and belongs to the oxidase dependent Fe transporter (OFeT) family (Debut et al. 2006). Ftr1p functions as an iron permease. Fet3p is a multicopper oxidase with three cupredoxin-like domains coordinating four copper ions. The Fet3p protein possesses ferroxidase activity, which is required for oxidation of Fe^2+^ during (or prior to) translocation of iron across the cytosolic membrane by the iron permease, Ftr1p. In addition, there are other homologous systems for the EfeUOB operon present in *Bacillus subtilis* and *Neisseria meningitides* (Baichoo et al. 2002; Grifantini et al. 2003; Ollinger et al. 2006), FtrABCD ferrous iron transporter in *Bordetella* species (Brickman and Armstrong 2012), and FetMP, a dipartite iron uptake system from *E. coli* strain F11 (Koch et al. 2011). The P19-cFtr1 iron uptake system is another identified example in *Campylobacter jejuni* (Chan et al. 2010) in which P19 is a 17 kDa periplasmic protein that together with cFtr1 (Ftr1p/EfeU homologue) is responsible for delivering iron to the cytosol. FepABC is another EfeUOB-like iron transport system present in *Staphylococcus aureus* (Turlin et al. 2013), which also has been well characterized.

The three proteins that comprise the EfeUOB iron-transport system in *E.coli* K-12 are; EfeU, EfeO and EfeB (Cao et al. 2007). EfeU, an inner membrane transporter consists of 276 amino acids with seven transmembrane regions and belongs to the oxidase-dependent Fe transporter OFeT family (Debut et al. 2006). EfeU is a homologue of Ftr1p of yeast with an iron permease function during metal transport (Grosse et al. 2006). The second one, EfeB is of periplasmic origin and is secreted by the Tat pathway (Sturm et al. 2006). It is the only known haem-containing Tat substrate (Sturm et al. 2006) and belongs to the DyP peroxidase superfamily (Sugano 2009). The biochemical studies reported to date suggests diverse set of functions for EfeB which include a peroxidase role (Liu et al. 2011; Sturm et al. 2006) or as heme/PPIX binding proteins involved in catalyzing the removal of iron from heme preserving the tetrapyrrole ring intact (Letoffe et al. 2009). The third protein, EfeO is a 38 kDa periplasmic protein and is an apparent Sec substrate, with a role in Efe-mediated iron uptake (Cao et al. 2007). Despite this wealth of information, there remain gaps in our understanding of the functional role of EfeO proteins, in iron transport of EfeUOB/M systems in bacteria.

Our previous sequence-structure bioinformatics analysis of EfeO from *E. coli* K-12 highlighted some key features (Rajasekaran et al. 2010b). The domain topology studies suggested a two-domain organisation for EfeO with an N-terminal cupredoxin-like (Cup) domain, together with a C-terminal peptidase-M75 (M75) domain, accounting for almost the entire polypeptide. The gene-context studies for the M75 domain in EfeO also indicated the presence of two forms of EfeO protein; EfeO_I_ consisting of both Cup and M75 domains together and EfeO_II_ consisting of only an M75 domain and lacking a Cup domain. Such M75 only EfeO-like proteins are herein designated as ‘EfeM’ (Rajasekaran et al. 2010a). Here we report the X-ray structure determination and bioanalytical characterisation of EfeM_Psy_ from *Pseudomonas syringae* pv. *syringae* B728a; an EfeM family member that shares ~ 60% sequence identity with EfeO of *E. coli* K-12 (Rajasekaran et al. 2010a) in an effort to understand the role of this protein family member in the EfeUOB/M systems in bacteria.

This is the first structural report for an EfeM component, belonging to the *Pseudomonas syringae* EfeUOB/M iron-transport system. Firstly, our structural data reveals a bi-lobate architecture for EfeM_Psy_ that shares similarities with the structural fold seen in bacterial periplasmic substrate/solute binding proteins (pSBP) (Berntsson et al. 2010; Felder et al. 1999; Fukami-Kobayashi et al. 1999; Scheepers et al. 2016; Tam and Saier 1993) and may be relevant to its function. Secondly, the crystal structure has allowed a more detailed investigation of the amino acid ligands contributing to Site III and Site IV of EfeM_Psy_, likely involved in metal binding. Thirdly, the SRCD titration assays, ICP-OES metal saturation studies, and in silico GRID analysis sheds light on the probable metal-binding specificities of EfeM_Psy_. Collectively, these observations help pave the way toward a more detailed understanding of the importance of peptidase-M75 domains for iron transport functions in EfeO/M proteins of the EfeUOB/M iron uptake systems in bacteria.

## Materials and methods

### Materials

The chloride salts of all metals used for preparing the metal stock solutions were purchased either from Sigma or Fisher (minimum 99% purity). The buffers for all structural and metal binding assays were prepared with Milli-Q water. The protein samples, at various stages of purification, were thoroughly dialysed, using dialysis membrane tubing of molecular weight cut off (MWCO) 12,000 Da from Medicell International Limited. The protein samples were concentrated, based on the specific requirements for each experiment, using Vivaspin concentrators (Sartorius Ltd) of 10,000 Da MWCO, according to the manufacturer’s instructions*.*

EfeM_Psy_ from *P. syringae* pv. *syringae* (over-expressed in *E. coli* K-12 with no affinity tags, as previously reported (Rajasekaran et al. 2010a)) was used for all biochemical and structural characterisations in this study.

### Crystallisation, data processing and structure determination of EfeM_Psy_

The protocols for generating apo-EfeM_Psy_ crystals, followed by X-ray diffraction data collection and data processing statistics have been described elsewhere (Rajasekaran et al. 2010a). Details of attempts to obtain a metal bound complex can be found in the Supplementary Methods. The structure of EfeM_Psy_ was solved by molecular replacement, using the PHASER program implemented in the CCP4 package (Winn et al. 2011). A cell-surface alginate-binding protein, Algp7 from *Sphingomonas* sp. A1 (PDB code: 3AT7) (Hashimoto et al. 2009; He et al. 2008; Maruyama et al. 2011) was used as the search model template. Initially, a theoretical model for EfeM_Psy_ (amino acids 35–285) was constructed based on the alignment with amino acid residues 25–275 of Algp7 (the model template), using the ‘CHAINSAW’ module from the CCP4 package. The corresponding theoretical model of EfeM_Psy_ from CHAINSAW was then used as the search model to look for possible packing solutions for EfeM_Psy_ using PHASER. Refinement of the EfeM_Psy_ structure solution was carried out using PHENIX (Adams et al. 2010). The refinement steps included initial rounds of rigid body and simulated annealing, followed by real-space refinement. Manual model building, during the iterative refinement steps, was accomplished using COOT (Emsley and Cowtan 2004). Water molecules were added both manually and automatically (using PHENIX) to the structure solution, where appropriate. The validation of the final model was accomplished using Molprobity (Chen et al. 2010). The data collection and refinement statistics, generated using PHENIX, are provided in Table 1. The structure based alignments between EfeM_Psy_ and Algp7/imelysin-like family members were carried out using the PDBeFold server (Krissinel and Henrick 2004). The search for similar structures against EfeM_Psy_, as the search probe, was performed using the DALI server (Holm and Rosenstrom 2010). The electrostatic potential maps for all the protein structures used in this study were generated using the PDB2PQR server (Dolinsky et al. 2004) and, graphic representation and interpretation of the structures were performed using PYMOL (The PyMOL Molecular Graphics System, Version 1.7.4 Schrödinger, LLC.).

**Table 1:** Data collection, refinement and model quality statistics for native EfeM_Psy_ protein

PDB code	7Q1G (Native EfeM_Psy_)	
*Data collection statistics*		
Synchrotron beamline, wavelength (Å)	Diamond I02, λ = 0.9796	
Resolution range (Å)	36.80–1.60 (1.69–1.60)	
Space group	P 2 2_1_ 2_1_	
a, b, c [ Å]	46.80, 95.23, 152.75	
α, β, γ [°]	90.00, 90.00, 90.00	
Total reflections	318,187 (46,648)	
Unique reflections	90,374 (13,064)	
Multiplicity	3.5 (3.6)	
Completeness (%)	99.4 (99.6)	
Mean I/sigma (I)	10.6 (2.0)	
CC (1/2)	0.997 (0.668)	
R_merge_	0.071 (0.625)	
R_pim_	0.043 (0.384)	
R_meas_	0.083 (0.737)	
Solvent content (%)	60	
Molecules per asymmetric unit	2	
*Refinement and stereochemical parameters*		
Number of reflections (working + test set)	90,313 (8935)	
Number of reflections (test set)	4522 (416)	
R-work (%)	18.51	
R-free (%)	20.97	
Protein residues	502	
Number of atoms	4617	
*Macromolecules*	3897	
*Ligands*	50	
*Water*	670	
Ramachandran favoured (%)	98	
Ramachandran outliers (%)	0	
RMS from ideal (bond length) (Å)	0.005	
RMS from ideal (bond angles) (°)	0.71	
Average B-factor (Å)	24.4	
*Macromolecules*	22.6	
*Solvent*	34.9	
*Ligands* Molprobity Score	31.2 0.83	

The data is generated using ‘Table 1’ option and Refinement log files from PHENIX software

### EfeM_Psy_ metal binding site analysis by GRID-FLAP

The molecular interaction fields (MIFs) showing favourable interaction regions in EfeM_Psy_ were calculated using GRID within the FLAP (Fingerprints for Ligands and Proteins) programme suite (Baroni et al. 2007). GRID chemical probes (Goodford 1985) such as the ‘DRY’ probe, describing potential hydrophobic interactions, sp2 carbonyl oxygen ‘O’ and the amide ‘N1’ probes for the hydrogen-bond acceptor and donor potential, respectively, were used to locate and define the binding pocket of the target biomolecule (EfeM_Psy_ in this case). A GRID spacing of 0.5 Å was used. Similarly, following the pocket analysis, MIFs between EfeM_Psy_ and select metal probes (Zn^2+^, Cu^2+^ Fe^2+^, Fe^3+^), as ligands, were calculated to investigate the potential metal binding sites in EfeM_Psy._ The resulting GRID MIFs describe regions in the protein where the corresponding interacting atoms (in this case, the metal ion) would be favourably located.

### Metal binding analysis by inductively coupled plasma optical emission spectrometry (ICP-OES)

The detection of metals in the apo-and metal-incubated forms of purified EfeM_Psy_ was performed by ICP-OES, using the instrument PERKIN-ELMER Optima 3000. The metal stock solutions were prepared by dissolving the chloride salts of each metal tested in 30 mM MES, pH 6.0. In the case of ferrous iron, the stock solutions were prepared in 30 mM MES, pH 5.0, to reduce oxidation of the ferrous iron. The proteins (2–50 µM) were incubated with 0.25 mM metal stock solution separately (Cu^2+^, Zn^2+^, Fe^2+^, Mg^2+^, Mn^2+^ and Fe^3+^) at 4 °C for 1 h. The unbound metals were then removed by subjecting the incubated samples to dialysis, using 12 kDa MWCO dialysis tubing against metal-free buffer (30 mM MES, pH 6.0 or pH 5.0) with the replacement of buffer every 8 h three to four times to remove the unbound metals. In the case of the Fe^2+^ binding experiments, to prevent oxidation the buffer pH was maintained at 5.0 and nitrogen gas was bubbled/purged into the dialysis system at regular intervals.

### EfeM_Psy_ metal titration studies by synchrotron radiation circular dichroism (SRCD)

The SRCD spectra for EfeM_Psy_–metal titration studies were recorded at 25 °C on Beamline B23 at Diamond Light Source. Purified protein, at a concentration of 10 µM in 20 mM HEPES pH 7.4 was used for recording the spectrum of the titration experiments. For titration studies, the SRCD spectra were measured with a 0.5 mm pathlength, Suprasil quartz cell (Hellma®) in the Far-UV CD region (180–260 nm) with a bandwidth of 1 nm and scan speed of 20 nm/min. A 1–10 µl aliquot of metal stock solution (for each metal such as Cu^2+^, Zn^2+^, Fe^3+^ tested) was added stoichiometrically to the protein solution, until the protein reached saturation, as followed spectroscopically. The raw SRCD spectral data in ellipticity (θ) as milli-degrees collected for Far-UV region (180–260 nm), obtained from either four or eight scans, were further processed and finally expressed in molar circular dichroism (Δε) using average amino acid residue molecular weight of 113 daltons.. The dissociation constant (K_d_) and stoichiometry of binding were calculated from the plot of ‘∆A’ Absorbance data points (reported as ∆A = A_L_−A_R_) at 222 nm against the metal (Zn^2+^, Cu^2+^) concentration. The fitting of SRCD data points was carried out using Hills Equation (Siligardi et al. 2002) with CDApps software (Hussain et al. 2015).

## Results and discussions

### Structure determination and model quality

In previous work, we successfully produced pure EfeM_Psy_ followed by single, diffraction quality crystals and data collection to high-resolution (1.6 Å) for EfeM_Psy_ (Rajasekaran et al. 2010a). Herein, we report a structure solution of the apo form of EfeM_Psy_ by molecular replacement.

The mature form of EfeM_Psy_ consists of 251 residues (Rajasekaran et al. 2010a). All 251 residues (aa 35–285) were localised in the final refined structure. The refined structure of EfeM_Psy_ consists of two identical EfeM_Psy_ monomers (chain A, residues A35-A285, and chain B, residues B35-B285) in the asymmetric unit. The R_work_/ R_free_ values for the EfeM_Psy_ structure are 18.51/20.97%, respectively (Table 1). A Ramachandran plot of the final structure shows 98.0% of residues in the most favoured regions, with no residues in the disallowed regions suggesting a stereochemically acceptable structure. There are no irregularities in the stereochemical parameters such as bond lengths, angles and torsion distributions for main- and side-chains, or distorted geometry for the final refined structure (Table 1) based on a Molprobity analysis (Chen et al. 2010).

### Overall structure

**Fig. 1:**
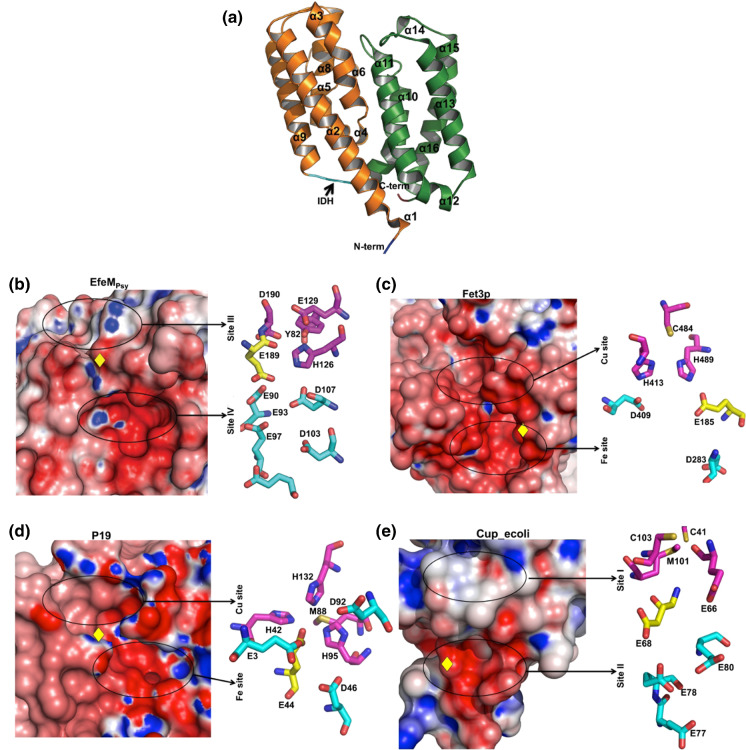
Crystal structure of EfeM_Psy_ from P*. syringae* pv. *syringae* (PDB code: 7Q1G) and its comparison with periplasmic iron transport components from representative partially related EFeUOB systems. **a** Overall structure of EfeM_Psy_ showing two helix bundle domains ‘B1’ and ‘B2’ coloured light orange and green, respectively, with inter-domain hinge (IDH) loop in cyan; **b** Electrostatic surface representation highlighting metal-binding pockets and amino acids lining Site III and Site IV of EfeM_Psy_. As in EfeM_Psy_, the other related proteins from various EfeUOB-like systems, namely; **c** Fet3p; Fet3p from *S. cerevisiae* (PDB code: 1ZPU); **d** P19 protein from *C. jejuni* (PDB code: 3LZQ); **e** Homology model for cupredoxin domain; Cup_ecoli (Cup-I family) of EfeO from *E. coli* (Rajasekaran et al. 2010b), all showing a two metal site network consisting of a copper site/Site I, coupled to an acidic patch with Glu/Asp ligands coordinating Mn^2+^ (as an iron analog) or Fe^2+^/Fe^3+^. The residues lining Site I/Site III/Cu and Site IV/Fe site are highlighted in magenta and in cyan (ball and sticks), respectively. The approximate location for the Asp residue situated at the interface between these two sites is highlighted as a yellow diamond in the electrostatic surface representations. The black ovals show the location of these metal sites. For **b**, **c**, **d** and **e**, the first part of the figure represents the electrostatic potentials for the respective protein, followed by representation of the metal site amino acid ligands (as ball and sticks)

Each EfeM_Psy_ monomer is an alpha-helical structure with 16 helices (designated as ‘α’). Of the 16 helices, twelve are α-helix and the remaining four (α1, α6, α7, and α14) are 3_10_ helices (Fig. 1a). In addition, 10 β-turns and 1 γ-turn are present. There are no β-sheet secondary structures. All secondary structure assignments are based on DSSP (Kabsch and Sander 1983). Close examination of the overall structure shows that EfeM_Psy_ is composed of two major α-helical bundles designated as ‘B1’ and ‘B2’. A flexible loop (residues 162–165, highlighted as ‘IDH’ in Fig. 1a) separates the B1 (residues 36–161) and B2 bundles (residues 166–285). The secondary structure composition shows 64% of amino acids in EfeM_Psy_ are in helices and the remaining 36% comprise either 3_10_ helices, turns or coils. A pairwise Cα structural superimposition analysis, using the DALI server, showed that both monomers (chain A and chain B) are similar to each other with an RMS deviation of 0.5 Å.

The overall structural organisation of EfeM_Psy_ is reminiscent of that generally observed in pSBP proteins. In general, pSBPs consist of two globular domains, which are comprised of mixed α/β structures and connected by an inter-domain hinge region consisting of either two or three β strands, or one long inter-connecting α-helix that tethers the two domains (Fig. S1a, b, c). Comparison of the folds (EfeM_Psy_ vs pSBPs) suggests that some of the key structural characteristics of pSBPs are retained by EfeM_Psy_; the presence of two discrete domains (Fig. S1d) linked by a hinge region, and the presence of an inter-domain ligand-binding cleft (Fig. S1d). However, as a member of the EfeM family, EfeM_Psy_ differs from other pSBPs such that it is largely α-helical. Moreover, the inter-domain hinge region of EfeM_Psy_ is smaller, consisting of only four residues which may reflect the relative size of ligand, in this case a metal ion, and potentially the lack of necessity to undergo any substantial opening/closing of the cleft to facilitate ligand binding.

### Structural comparison of EfeM_Psy_ with other proteins using DALI server and PDBeFold

A search for similar structures of EfeM_Psy_ was performed using the DALI server, submitting the EfeM_Psy_ structure as the probe. The search results yielded various neighbouring protein entries from different families, showing Z-score ranging between 45 and 2.0 with diverse functions. The top hit members (except EfeM_Psy_) with Z-score > 21 were from Algp7 and imelysin-like protein family members, possessing a peptidase-M75 domain. For the purpose of clarity, the comparisons involving both Algp7 and imelysin-like proteins are designated as ‘Algp7/imelysin-like family’ throughout the remaining text. With few exceptions, all other remaining structure hits with Z-score (< 7.5) did not belong to either Algp7/imelysin-like family or the EfeO_I_/EfeO_II_ family. Among the top hit members, Algp7 protein was an alginate binding protein (Maruyama et al. 2011; Temtrirath et al. 2015) from *Sphingomonas sp*. A1 (strain A1), an alginate-assimilating bacterium (PDB codes: 3AT7,3WSC and 6JBO). Further studies also reported this alginate-binding bacterial protein, Algp7, as an EfeO-like protein belonging to the EfeUOB-like operon in alginate-assimilating *Sphingomonas *sp. A1 (strain A1) species (Temtrirath et al. 2017).

Two other prominent imelysin-like protein family members were ‘IrpA’, a putative iron-regulated protein (PDB code: 4ECG; sequence identity = 20%) from *Parabacteroides distasonis* ATCC 8503 and ‘IPPA’, the second imelysin-like protein (Xu et al. 2011) (PDB code: 3PF0; sequence identity = 12%) from *Psychrobacter arcticum*. Among the few exceptions mentioned above, PIBO from *Bacteroides ovatus* ATCC 8483(Xu et al. 2011) (PDB codes: 3N8U and 3OYV), a well-known member of the imelysin-like protein family, appeared as a hit member with Z score of ~ 7.0. In summary, all top match members shared a common peptidase-M75 domain structural organisation with an alpha helical topology. A detailed structure comparison between EfeM_Psy_ and its structurally homologous Algp7/imelysin-like family protein structures, analysed using the PDBeFold server, shows that the peptidase-M75 fold of EfeM_Psy_ fits with other Algp7/imelysin-like family members with its distinctive two four-helix bundle domains with minor variations observed around the loops related to the two four-helix bundle domains (Fig. S2a and Fig. S3. It is interesting to note that the structural homology observed for EfeM_Psy_ with other similar protein hits (with low Z-score < 7.5), from the DALI homologous structure search, revealed the presence of four-helix bundles being the common structural motif observed in these proteins. At the same time, the likely reason for the diverse functions between these similar proteins, is due to the variations at the site of helix-helix, loop-loop and helix-loop interactions.

### Structural mapping of Site III and Site IV metal sites of EfeM_Psy_

**Fig. 2:**
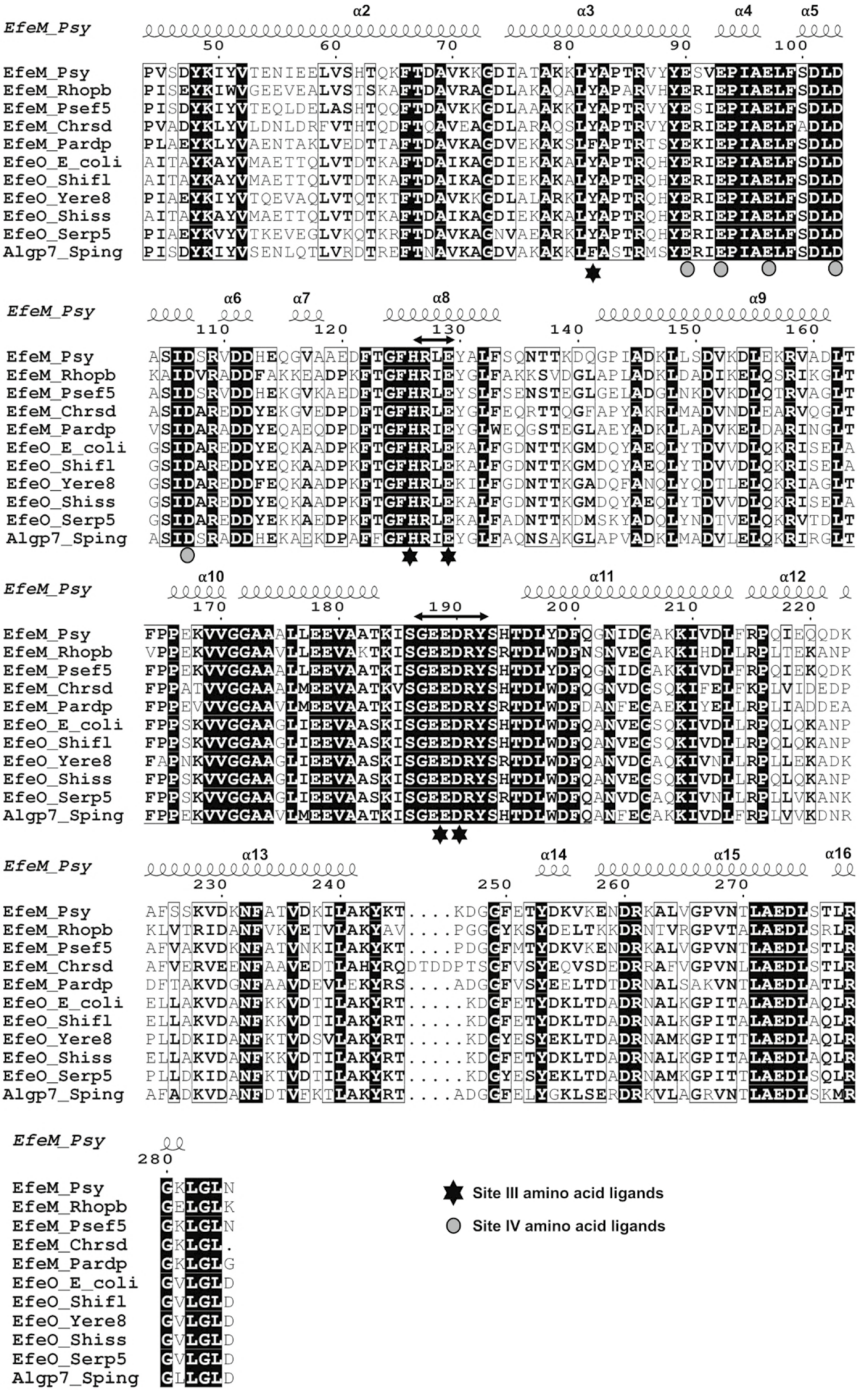
Multiple sequence alignment (MSA) for peptidase-M75 domain region of EfeM_Psy_ with selected homologues belonging to the EfeO (Cup domain with peptidase-M75) and EfeM (peptidase-M75 only) families. The search for representative homologues was performed using the gene name for EfeM_Psy_ (‘Psyr_3370’) as the search probe in the STRING database (Szklarczyk et al. 2019). The protein sequences, for both families, were found by this search and the MSA was calculated using the CLUSTAL OMEGA server (Sievers et al. 2011). Visualisation of the alignment was carried out using the ESPript server (Robert and Gouet 2014). For Site III, all five residues (Tyr82, His126, Glu129, Glu189, Asp190) are completely conserved whereas, similar residues Tyr/Phe and Asp/Glu align for positions 82 and 190, respectively. In the case of Site IV, all five residues (Glu90, Glu93, Glu97, Asp103 and Asp107) are completely conserved throughout the alignment. The ‘HXXE’ and ‘GEEDRY’ motifs are highlighted by double headed arrows. In addition, Algp7, a prominent member from Algp7-imelysin like family is also included in the alignment for comparing the metal site conservation between EfeO/EfeM and Algp7-imelysin like family. The UNIPROTKB database accession numbers for the representative entry sequences are listed out in the Supplementary Table S1

Our previous sequence-based bioinformatics study of the EfeM/EfeO family suggested the occurrence of the peptidase-M75 domain in the EfeO_I_/EfeO_II_ family with a highly conserved 'HXXE' motif. In this work, a closer examination of the structure of EfeM_Psy_ has revealed two potential metal binding sites. The two sites are designated ‘Site III’ and ‘Site IV’. The metal binding cleft, Site III, is nestled in between the two alpha helix bundles B1 and B2. In addition to the HXXE motif for Site III (Fig. 1b), this site also could include one/two amino acids from the 'GEEDRY' motif loop (residues 187–192), which are highly conserved among EfeO/EfeM family members (Fig. 2). Glu189 and Asp190 (of 'GEEDRY' motif) and Tyr82 sit in the vicinity of this Site III binding motif (Fig. 1b). Based on these observations, arising from the crystal structure, up to five residues (Tyr82, His126, Glu129, Glu189 and Asp190) can be considered potential metal ligands at Site III (Fig. 1b).Assessment of the structure for other potential metal binding sites, suggests a potential second metal binding site for these EfeO/EfeM family of proteins. This additional potential metal binding site is designated 'Site IV' (Fig. 1b). This site is highly acidic, comprised primarily of Glu and Asp residues, which appear to form a binding pocket adjacent to Site III. These acidic residues are comprised of residues from bundle B1 and B2 (Glu90, Glu93, Glu97, Asp103 and Asp107). Glu 179 also may contribute to binding Site IV. Moreover, all of these acidic residues are highly conserved, as shown in the multiple sequence alignment of the peptidase-M75 domains of EfeM/EfeO family proteins (Fig. 2). The metal binding potential for Site IV of EfeM_Psy_ is supported by the findings observed in the Algp7-Cu^2+^ complex crystal structure (PDB code: 5Y4C; (Temtrirath et al. 2017). The key amino acid ligands lining the Cu^2+^ site of Algp7 also appear to be Glu/Asp enriched (Glu79, Glu82, Asp96 and Glu178 of Algp7) similar to those observed in lining the Site IV of EfeM_Psy_ (Fig. 2 and Fig. S4). Similarly, crystal structure for IrpA (PDB code:4ECG), another Algp7-imelysin like family member also reported a calcium (Ca^2+^ at position 504) bound in the vicinity of Glu/Asp enriched pocket lined by residues Glu111, Glu114, Asp131 and Glu299 of IrpA (Fig. S4). This binding cleft, observed in IrpA, structurally aligns with our proposed Site IV cleft of EfeM_Psy_. Moreover, Glu111, Glu114, Asp127 and Asp131 of IrpA are 100% conserved (in both sequence and structure) with our proposed residues Glu90, Glu93, Asp103 and Asp107 of Site IV of EfeM. Moreover, both sites (Site III and Site IV) are solvent exposed and, as such, allow water-mediated interactions to further support metal binding to the protein. In summary, all these observations are suggestive of a potential metal binding role at Site IV in EfeM_Psy_, which may contribute to its iron transport function.

### Structural comparison of metal sites of EfeM_Psy_ with periplasmic iron transport components from representative partially related/homologues EfeUOB systems

Metal site (Site IV) comparison studies between EfeM_Psy_ and other periplasmic iron transport components of partially related/homologues EfeUOB systems gives rise to some noteworthy observations. In the case of Fet3p (from the Ftr1p-Fet3p system), there is an acidic patch of amino acid residues, Glu185, Asp283 and Asp409, located adjacent to the T1/Cu site, within a distance of ~ 5 Å of each other (Fig. 1c). For P19 (from the P19-cFtr1 system), the “Mn-P19” crystal structure (PDB codes: 3LZQ and 3LZR) shows an acidic patch, also consisting of Asp and Glu residues (Glu3, Glu44, Asp46 and Asp92), holding a Mn^2+^ atom (as a likely iron-binding site) (Fig. 1d), which is located at distance of ~ 7 Å from the Cu^2+^ site. Similarly, from our previous work (Rajasekaran et al. 2010b), Site II in the Cup_ecoli_ homology model (Fig. 1e) from *E. coli* is composed of four Glu residues (Glu68, Glu77, Glu78, Glu80) and lies ~ 12 Å from the Site I/Cu site. Site II is well conserved and shows preference for ferric iron (Rajasekaran et al. 2010b).

Comparison with the putative iron-binding sites of Fet3p, P19, and Cup_ecoli_ (Fig. 1) suggests that EfeM_Psy_, with its acidic patch adjacent to its divalent site located in proximity to Site III (~ 9 Å away), likewise may be important for iron binding. A preference for ferric and/or ferrous iron for EfeM_Psy_ is supported by our ICP-OES (Table 2). The metal site comparative analysis highlights a plausible role for Site IV, analogous to that of Site II in Cup_ecoli_ or the Fe site of Fet3p and P19, acting as a transient site to hold the ferric iron prior to transfer to EfeU for ultimate transport across the membrane, following initial capture and/or ferroxidation by the other components.

**Table 2:** Metal content analysis for the ‘as isolated’ and ‘metal incubated’ forms of EfeM_Psy_

Element	Metal salts^c^	‘As isolated’	‘Metal-incubated’
		Metal/protein stoichiometry	Metal/protein stoichiometry
Cu^2+^	CuCl_2_	0.019 ± 0.017	1.572 ± 0.125
Fe^2+^ and Fe^3+^	Total iron	0.011 ± 0.003	0.00^b^
Fe^2+^	NH_4_)_2_Fe(SO4)_2_·6H_2_O	0.00^a^	1.446 ± 0.302
Fe^3+^	FeCl_3_	0.00^a^	1.327 ± 0.226
Zn^2+^	ZnCl_2_	0.027 ± 0.007	0.451 ± 0.031
Mg^2+^	MgCl_2_	0.042 ± 0.005	0.021 ± 0.007
Mn^2+^	MnCl_2_	0.001 ± 0.000	0.000 ± 0.00

^a^The total iron content is described in the case of ‘as isolated form’

^b^The iron content is described separately as ‘Ferrous’ and ‘Ferric’ for ‘metal-incubated form’

^c^The corresponding metal salts used to make element stock solutions for preparing EfeM_Psy_-metal-incubated forms

A final observation, from a comparison of Site IV of EfeM_Psy_ to P19, Fet3p (Fig. 1) and Algp7/imelysin-like members (Fig. S2), is the well conserved glutamic acid Glu189 of EfeM_Psy_ that lies at the interface between Site III and Site IV, which may be important for the concerted function of these two sites (Fig. 1b). The importance of this residue is worth further investigation.

### Structural comparison of metal sites of EfeM_Psy_ with Algp7/imelysin-like family member proteins

Comparison of the metal binding site motifs of EfeM_Psy_ to other Algp7/imelysin-like family members such as Algp7, IrpA, PIBO (Fig. S2b, c, d) shows that the amino acid ligands His126 and Glu129 (Fig. 1b), lining Site III ('HXXE' motif), are structurally conserved in all proteins, except IPPA (Fig. S2e), which has a proline at position 126 replacing the otherwise conserved histidine. The other two potential acidic residues, Glu189 and Asp190 in Site III ('GEEDRY' motif loop), observed in this study, are either conserved or replaced with similar residues, again for all proteins except IPPA (Fig. S2e). In the case of IPPA, it lacks any conserved/similar residues (Pro286 and Ala287) that would map onto the 'GEEDRY' motif loop. The last amino acid ligand of Site III, Tyr82, which also could contribute to metal binding, is either fully conserved or replaced with a Trp residue. As reported in Fig. 2 and Fig. S4, the amino acid ligands in Site IV for EfeM and Algp7/imelysin-like family members are either fully conserved or similar for all four proteins except IPPA. IPPA also displays a different pattern of amino acid ligands lining Site IV, with very few residues fully conserved.

### In silico predicted metal site binding at Site IV of EfeM_Psy_ by GRID-FLAP

**Fig. 3:**
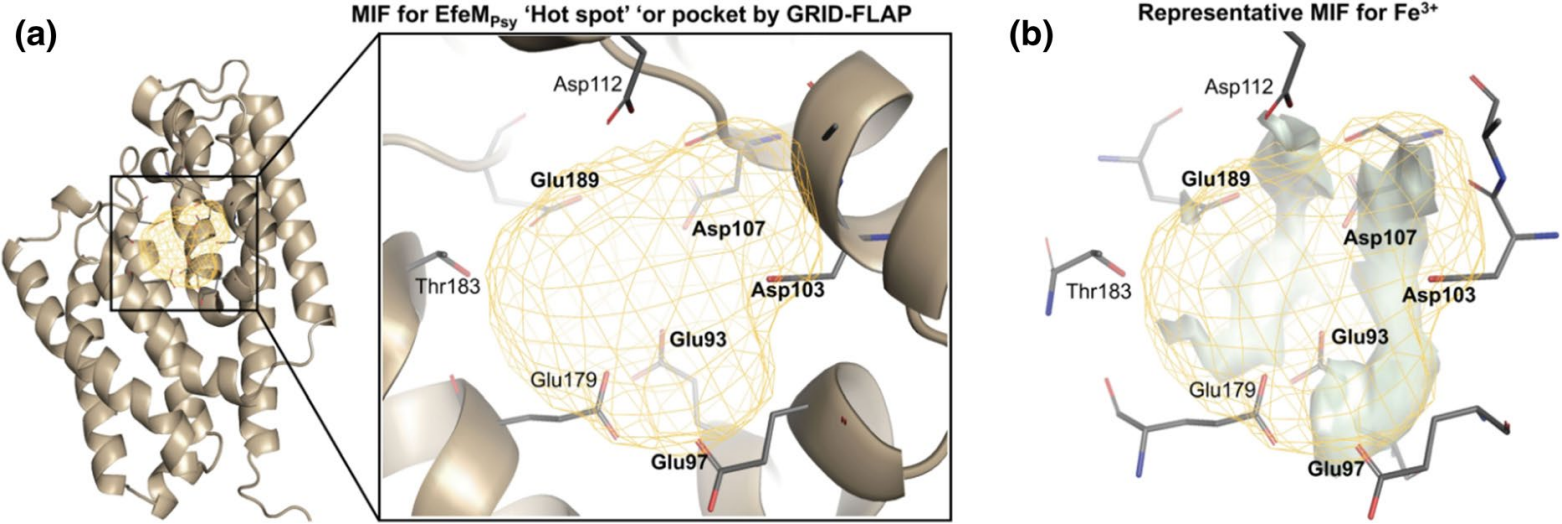
Characterisation of the EfeM_Psy_ metal binding Site IV and its interaction with select metals by GRID-FLAP. **a** GRID molecular interaction field (shown as gold contour) in the region of our proposed metal binding site, Site IV for EfeM_Psy_, highlights the key Glu/Asp amino acids lining the binding pocket. Glu90 also is identified as a potential ligand by GRID-FLAP, however, it is not displayed here in this orientation, which displays the best possible close-up view of the MIF contours **b** Representative GRID molecular interaction fields, shown as three-dimensional contours (solid grey regions), shows specific favourable regions of EfeM_Psy_ for interaction with Fe^3+^ with the highest MIF energy of − 81.58 kcal/mol suggesting Fe^3+^ as the most favoured metal followed almost equally by the other select metals Fe^2+^, Cu^2+^, Zn^2+^ with MIF energies of − 54.26, − 54.17 and − 53.43 kcal/mol, respectively. The three-dimensional constellation of amino acid residues permits favourable six coordinate geometry for metal binding

Using the program FLAP, to calculate GRID molecular interaction fields (MIFs), an additional binding pocket was found for EfeM_Psy_ (Fig. 3a). The pocket revealed that most of the residues lining this putative binding pocket (Fig. 3a) belonged to our identified Site IV, possessing a Glu/Asp amino acid core (Glu90, Glu93, Glu97, Asp103, Asp107). In addition, the pocket consisted of other Glu residues (Glu189 and Glu179) that also could play a role in metal binding. GRID MIFs were also calculated for select metals Zn^2+^, Cu^2+^, Fe^2+^ and Fe^3+^ (which indicated the most favourable interaction region of EfeM_Psy_ where potential binding of these metals would be predicted. In each case, the binding site is filled by residues from the putative Site IV metal site. The representative MIF for EfeM_Psy_–Fe^3+^ (Fig. 3b) showed the highest MIF energy of − 81.58 kcal/mol suggesting that Fe^3+^ would be the most favoured metal ligand, followed almost equally by the other metals based on the MIF energies (see Fig. 3 figure legend details).

Crystal structures of EfeM_Psy_-metal complexes would have provided a more definitive understanding of the precise metal binding region of EfeM_Psy_ and the key amino acid ligands involved. To achieve this, metal soaking and co-crystallisation experiments of EfeM_Psy_ with various metals (Cu^2+^, Zn^2+^, Fe^2+^, Fe^3+^, Mg^2+^ and Mn^2+^) were attempted (see Supplementary Methods for further details). However, we were unable to obtain any promising results despite numerous attempts. Under the conditions required to obtain diffraction quality crystals, we observed a few sulphate ions in our structures (resulting from lithium sulphate and/or ammonium sulphate buffer components) and, importantly, we observed a few non-specific sulphate ions located in the vicinity of our proposed Site III/IV pocket thus, there is the possibility that this could have precluded metal binding, at the concentrations used. Nevertheless, in the absence of a metal bound crystal structure, our GRID analysis supports the additional Site IV metal site and independently identifies the same binding site residues that correspond to those identified in our sequence-structure analysis.

### ICP-OES analysis confirms that EfeM_Psy_ is a metal-binding protein

The structure of EfeM suggests the presence of two potential metal-binding sites (designated Site III and Site IV). To further explore the metal-binding capacity of EfeM_Psy_, the protein was incubated with excess concentrations of the metals of interest, followed by removal of excess metal by dialysis and then quantitative analysis of the remaining bound metal, using ICP-OES.

ICP-OES showed that the ‘as isolated’ EfeM_Psy_ protein, after purification and before metal treatment, is essentially metal free, with levels well below stoichiometric values at 0.01–0.05 ions/ EfeM_Psy_ molecule (Table 2). Following independent metal incubation and subsequent dialysis, against metal-free buffer, several metals were found to be retained by EfeM at levels indicative of specificity (Table 2). Cu^2+^, Fe^2+^ and Fe^3+^ each were found associated with EfeM_Psy_ (Table 2) at a molar stoichiometry of ~ 1.5 metal ions per EfeM_Psy_. This would be consistent with the possibility for two partially occupied EfeM binding sites for these transition metals, possibly corresponding to Sites III and IV. It is likely that the iron added to EfeM as Fe^2+^ would convert to the oxidised Fe^3+^ state, over the course of the experiment, indicating that a similar amount of ferric iron remains associated with EfeM no matter whether ferrous or ferric iron is initially provided. Zn^2+^ was retained by EfeM at a stoichiometry of ~ 0.5:1, which indicates at least one Zn^2+^ binding site for EfeM, which must be within the peptidase-M75 domain, again most likely to be Site III. No significant binding was observed for the other metals tested (Mg^2+^, Mn^2+^). Overall, the metal incubation studies of EfeM_Psy_ clearly indicate a capacity for tight binding to Cu^2+^, Zn^2+^, Fe^2^ and Fe^3+^, but not to Mg^2+^ and Mn^2+^. Taken together, ICP-OES data showed that EfeM_Psy_ are associated with metals, but the precise locations and preferences/specificity of metals bound remain to be determined.

### Synchrotron-radiation circular-dichroism analysis supports EfeM_Psy_ metal binding

The far-UV SRCD spectrum for apo EfeM_Psy_, reported in our previous studies (Rajasekaran et al. 2010a), displayed characteristic spectral features for proteins possessing a high content of α-helix. These results are in agreement with the secondary structure composition, as found in the EfeM_Psy_ crystal structure.

**Fig. 4:**
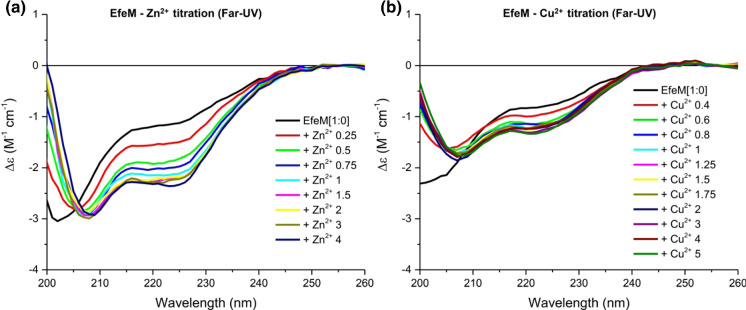
Far–UV (200–260 nm) SRCD spectra of apo EfeM_Psy_ and its titration with select metals. The SRCD spectrum of EfeM_Psy_ (180 μl of pure protein, ~ 10 μM, ~ 0.3 mg/ml) with metals was measured in 0.5 mm path-length Suprasil quartz cells (Hellma®) in the far-UV region (180–260 nm). Each spectrum is the average of four or eight scans expressed in molar circular dichroism (Δε) using average amino acid residue molecular weight of 113. For the purpose of clarity, the titration spectral data between 200 and 260 nm are presented. All the experiments were performed at 25 °C. **a** Left panel shows SRCD spectra of EfeM_Psy_ with Zn^2+^ additions with molar stoichiometry starting from 0.25 (2.5 μM) to 4 (40 μM); **b** Right panel shows SRCD spectra of EfeM_Psy_ with Cu^2+^ additions with molar stoichiometry starting from 0.4 (4 μM) to 5 (50 μM)

The metal-binding capacity of EfeM was analysed by monitoring changes in the far-UV spectral range upon titration of metals with EfeM_Psy_. The gradual titration of Zn^2+^ resulted in a proportional alteration of the spectrum until a molar stoichiometry of ~ 1:1 Zn^2+^:EfeM_Psy_ was achieved (Fig. 4a). No further major changes in the spectrum were observed as Zn^2+^ levels were raised from 1 (10 µM) to 4 (40 µM) molar equivalents. The spectral changes were most pronounced in the spectral region 216–225 nm from the Zn^2+^-dependent quench curve (Fig. 4a). Similar spectral changes were observed in the EfeM_Psy_–Cu^2+^ titration studies (Fig. 4b). The quench curve shows a dose-dependent change in the CD spectrum as either Zn^2+^ or Cu^2+^ were added, which reaches a plateau upon saturation and thereby demonstrates a binding event. These results suggest that Zn^2+^ and Cu^2+^ have a binding affinity to EfeM_Psy_ with the K_d_ determined to be 3.4 and 4.1 µM (Fig. S5), respectively. The stoichiometry plot for both metals (Zn^2+^ and Cu^2+^) indicated formation of an EfeM_Psy_–Cu^2+^/Zn^2+^ complex with 1:1 protein-metal stoichiometry, respectively (Fig. S5). No significant changes in the SRCD spectrum (region 216–225 nm), upon the addition of Fe^3+^, until a molar stoichiometry of ~ 5:1 Fe^3+^:EfeM_Psy_, were observed (Fig. S7)), however, the reason for this is unclear. By contrast, the SRCD titration attempts of EfeM_Psy_ with Fe^2+^ were inconclusive. It may be that, due to the required SRCD experimental conditions (buffer mediated factors, aerobic environment), such factors effected an authentic Fe^2+^ titration. Nevertheless, the SRCD-EfeM_Psy_ titration studies support a role for divalent metal binding to EfeM and the most likely location for this binding could be Site III, as supported by the crystal structure. It is also feasible to expect that the binding observed for Zn^2+^ and Cu^2+^ is representative of how EfeM_Psy_ might respond to Fe^2+^ at Site III, in its natural environment.

### Proposed functional roles of the metal binding sites of M75 domain in EfeUOB/M iron transport systems

Our structural studies, coupled with metal-binding assays and in silico GRID studies, presented herein, emphasize the structural features and metal binding potential of EfeM_Psy_. The EfeM_Psy_ crystal structure, possessing a bi-lobal fold, suggests structural similarities to pSBP. Our ICP-OES and SRCD studies support the EfeM_Psy_ potential metal binding capacity. Specifically, the binding affinity and stoichiometry for Zn^2+^ and Cu^2+^ with EfeM_Psy_, from the SRCD studies, supports the preference of EfeM_Psy_ for divalent cations (presumably ferrous iron, in this case, potentially through Site III). Additionally, our in silico GRID analysis highlights Site IV as a favourable binding pocket, postulating a potential metal binding role with its Glu/Asp rich amino acid ligands.

**Fig. 5:**
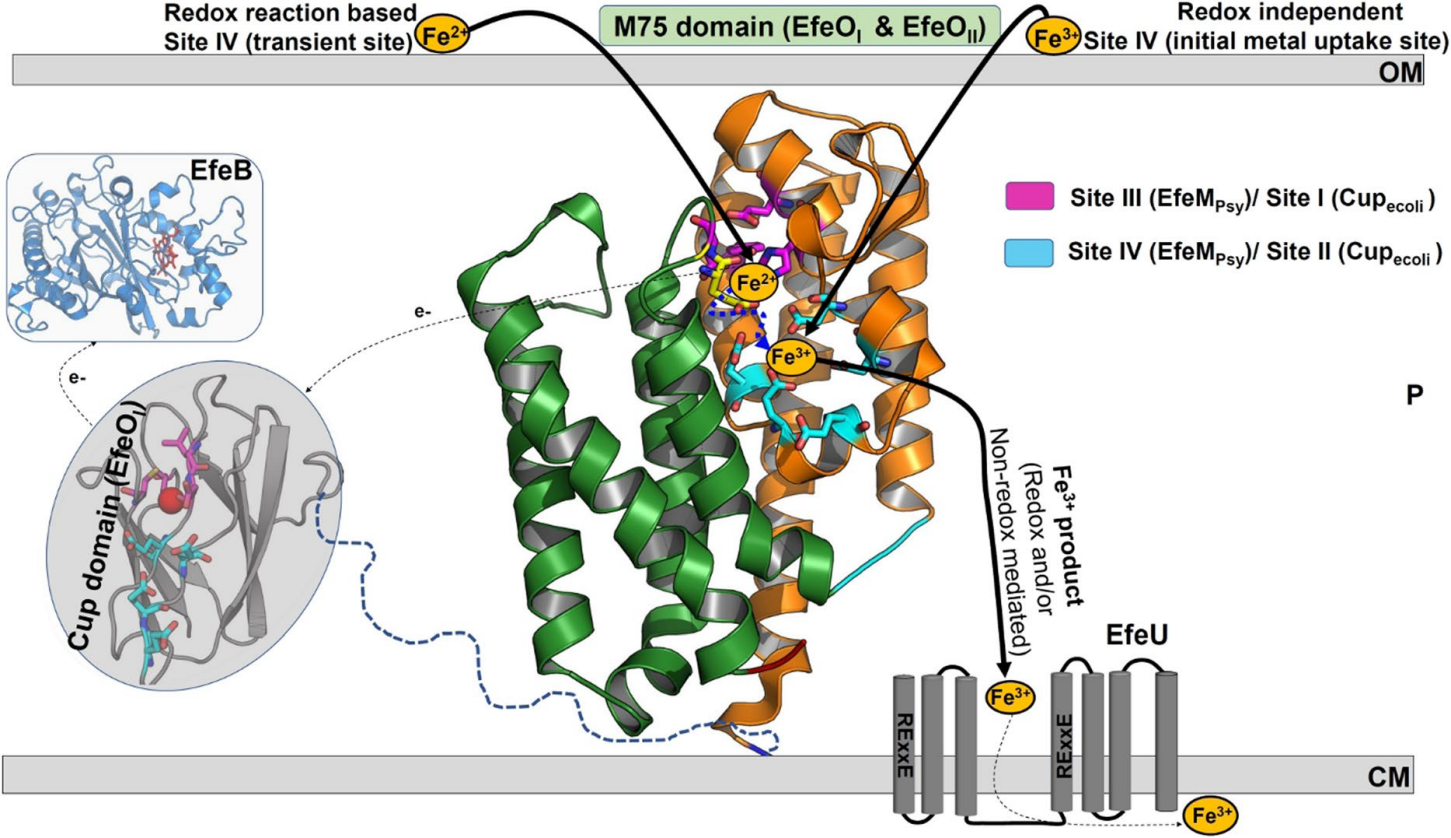
Hypothetical functional schema for the engagement of metal binding sites of M75 domain in EfeUOB/M iron transport systems. The four main components of EfeUOB/M system are EfeU, EfeO, EfeM and EfeB. EfeU is represented schematically on the cytoplasmic membrane (putative seven transmembrane helices are highlighted as grey cylinders with loop connections). Cartoon representation (blue colour) of periplasmic peroxidase, EfeB, our previously determined crystal structure with haem molecule represented as red sticks (PDB code: 2Y4F). The final protein EfeO/EfeM is represented by focusing the M75 domain of EfeM_Psy_ crystal structure (PDB code: 7Q1G). Our homology model for the cupredoxin domain of *E. coli* (28) is shown in grey cartoon with potential Cu^2+^ shown as red spheres. As part of hypothetical schema, M75 domain with its putative metal sites could assist in multiple roles according to the iron availability. The putative role of Site III initially for the uptake coupled with oxidation of ferrous iron or involved in direct uptake of ferric iron is a great possibility. Similarly, the involvement of M75 domain in translocating the Fe^3+^ product (generated either through EfeB mediated oxidation or by initial uptake of ferric iron from environment) to the cytosol through the EfeU component cannot be ruled out. In this case, proposed Site IV could play a possible role as transient Fe^3+^ holding site or initial Fe^3+^ uptake site. The selection of a suitable iron uptake pathway, comprising the best combinations of metal sites among these alternative routes, might be decided based upon the availability of iron in environment, its oxidation state and the need for iron acquisition. The subcellular locations are represented by following keywords: CM, cytoplasmic membrane; P, intermembrane space/periplasm; and OM, outer membrane. The putative ~ 20 amino acid linker between Cup and M75 domain in the case of EfeO_ecoli_ is shown as blue dash lines

In light of the data (see also^1^), a hypothetical functional schema for the engagement of the M75 domain, which could be applicable to both EfeO_I_ and EfeO_II_ protein family members in general, is described (Fig. 5). Keeping EfeM_Psy_ as the model template, a potential redox mediated involvement of the M75 domain for initial uptake of ferrous iron and/or oxidation, followed by ferric iron transport to the cytosol (mediated by Site III and Site IV), could be considered. In addition, a plausible role for the M75 domain (redox-independent) as an initial metal uptake site for ferric iron from the environment is still a possibility, involving the Site IV binding pocket. A similar kind of ferric iron preference for the EfeO component, ywbM from *Bacillus subtilis* (possessing only the M75 domain) is reported by Miethke et al. In their work, they demonstrated a working model for EfeUOB (ywbLMN) iron transport complex of *Bacillus subtilis* in which the binding protein EfeO and the permease EfeU form a minimal complex (EfeUO) for ferric iron uptake (Miethke et al. 2013). Taken collectively, this might suggest that M75 domains in EfeUOB/M systems might play a crucial role in iron uptake (either Fe^2+^ or Fe^3+^) through potential metal binding sites (Site III/Site IV), along with the Cup domain (Site I/Site II), according to the availability and necessity of different iron states.

The work reported here introduces EfeM_Psy_ as the best candidate to mark the beginning of the journey for a more detailed understanding of the periplasmic components, EfeO (EfeO_I_ family) and EfeM (EfeO_II_ family) and thereby offers support for the suggestion that these proteins contribute to iron transport in the EfeUOB(M) system, acting as periplasmic iron-binding proteins.

## Supplementary Information

Below is the link to the electronic supplementary material.

Supplementary file1 (DOCX 1766 kb)

## Footnotes

1 During the course of revisions for this manuscript, the crystal structure of EfeO from *Sphingomonas sp.* strain A1 (Nakatsuji, et al. Crystal structures of EfeB and EfeO in a bacterial siderophore-independent iron transport system, (2022), *BBRC*, 594, I24-I30) was published. In this work, the authors report a novel metal-binding motif “ExxE-//-D-//-E”, which incorporates elements of our newly identified metal binding site (Site IV), as revealed in this work. The authors also report stabilization of EfeO in the presence of Cu^2+^, Fe^2+^ and Zn^2+^ and present the structure of EfeO in the presence of a rare earth element (Sm^3+^) bound, which is shown to bind to highly conserved negatively charged residues at our Site IV (Glu90, Glu93, Asp103 and Asp107), identified herein. The authors also claim this novel metal-binding site, proposed as “ExxE-//-D-//-E” in their work, in the EfeO C-terminal M75 peptidase domain. Our work provides further support for this novel binding site in EfeM shown herein, which may in fact generalize this feature to M75 peptidases belonging to the EfeO_II_/EfeM family. At the time of submission, these newly reported structures were not available in the PDB database for further detailed comparisons.
