# Supplementary material for: Crystal structure and metal binding properties of the periplasmic iron component EfeM from Pseudomonas syringae EfeUOB/M iron-transport system
Source: Biometals. 2022 Mar 29;35(3):573–89. doi: 10.1007/s10534-022-00389-2 (PMC9174327; doi:10.1007/s10534-022-00389-2)
Supplement: Supplementary file 1 — Supplementary file1 (DOCX 1766 kb) [file 10534_2022_389_MOESM1_ESM.docx]

**Supporting information**

**Crystal structure and metal binding properties of the periplasmic iron component, EfeM from *Pseudomonas syringae* EfeUOB/M iron-transport system**

**Mohan B Rajasekaran^^[[1]](#footnote-2)^a^, Rohanah Hussain^b^, Giuliano Siligardi^b^, Simon C Andrews^a^, Kimberly A Watson^a^**

^a^School of Biological Sciences, Health and Life Sciences Building, University of Reading, Whiteknights Campus, Reading, RG6 6ES, UK;

^b^ B23 Beamline, Diamond Light Source, Harwell Science Innovation Campus, Chilton, Didcot OX11 0DE, UK.

**TABLE OF CONTENTS** **Page no**

Supplementary Methods S-2

**Figure S1**. Close-up view of the structural superimpositions, electrostatic surface S-3

representations and metal binding pocket comparisons of EfeM with other

Algp7/imelysin-like family members.

**Figure S2.** Comparison of putative Site IV binding pocket of EfeM_Psy_ with metal bound S-4

IrpA and Algp7 crystal structures.

**Figure S3:** Estimation of the binding affinity and stoichiometric calculations from

EfeM – Zn^2+^/Cu^2+^ SRCD titrations. S-5

**Figure S4.** Structure based comparison of EfeM_Psy_ with other pSBP structures. S-6

**Figure S5.** Overall structural superimpositions between EfeM and other

Algp7/imelysin-like family members; IrpA (PDB code:4ECG) and IPPA (PDB code:3PF0). S-7

**Figure S6.** Far–UV (200-260 nm) SRCD spectra of apo EfeM_Psy_ and its titration with Fe^3+^. S-8

**Table S1.** List of representative members from EfeO/EfeM and Algp7-imelysin like family S-9

**Supplementary Methods**

During the course of this work, significant efforts were made to generate X-ray crystal structures of EfeM_Psy_ with metal bound. EfeM_Psy_ – metal co-crystallisation experiments were carried out by addition of 1-10 fold molar excess of CuCl_2_, (NH_4_)_2_Fe(SO_4_)_2_·6H_2_O, FeCl_3_, MgCl_2_, MnCl_2_ or ZnCl_2_ stock solutions to the protein. The incubated/reconstituted EfeM_Psy_–metal samples were screened against commercially available crystal screens and apo EfeM_Psy_ optimised crystal screen condition (ammonium sulphate, lithium sulphate and 0.1 M Tris pH 7.0). In the case of iron, the infrastructure to carry out co-crystallisation experiments under strict anaerobic conditions were not available. Among our attempts, the iron stock solutions were kept at pH 5.0 and the inclusion of sodium dithionite to the protein samples was used to reduce the oxidation of iron. Similarly, Mn^2+^ also was tried in co-crystallisation experiments, as has been used in the literature by others, as an iron analog. Although we were successful in producing a few EfeM_Psy_ – Zn^2+^ co-crystals, the output of data processing revealed no bound Zn^2+^ in the structure. In the case of soaking experiments, the apo-EfeM_Psy_ crystals obtained (2.5 M ammonium sulphate, 0.1 Tris pH 7.0, 0.2 M lithium sulphate) were soaked in 1 to 5 fold molar excess of CuCl_2_, FeCl_3_, (NH_4_)_2_Fe(SO_4_)_2_·6H_2_O, MgCl_2_, MnCl_2_ or ZnCl_2_ solutions between 5-30 min. All the successful crystals, after soaking, were tested and the majority of the soaked crystals did not diffract well and collection of complete diffraction data sets for EfeM_Psy_- metal soaked crystals was not successful. It remains unclear to us, at the present time, as to why these attempts were unsuccessful, however, it might be the case that the presence of lithium sulphate and ammonium sulphate adversely affect the binding of such metals. We did observe a few sulphate ions in our structures (resulting from the buffer components) and, interestingly, we observed a few non-specific sulphate ions located in the vicinity of our proposed Site III/SiteIV pocket thus, there is the possibility that this might have precluded metal binding, at the concentrations used. This is speculation only and further experimental validation is required. At the very least alternative crystallisation conditions would be necessary.

**
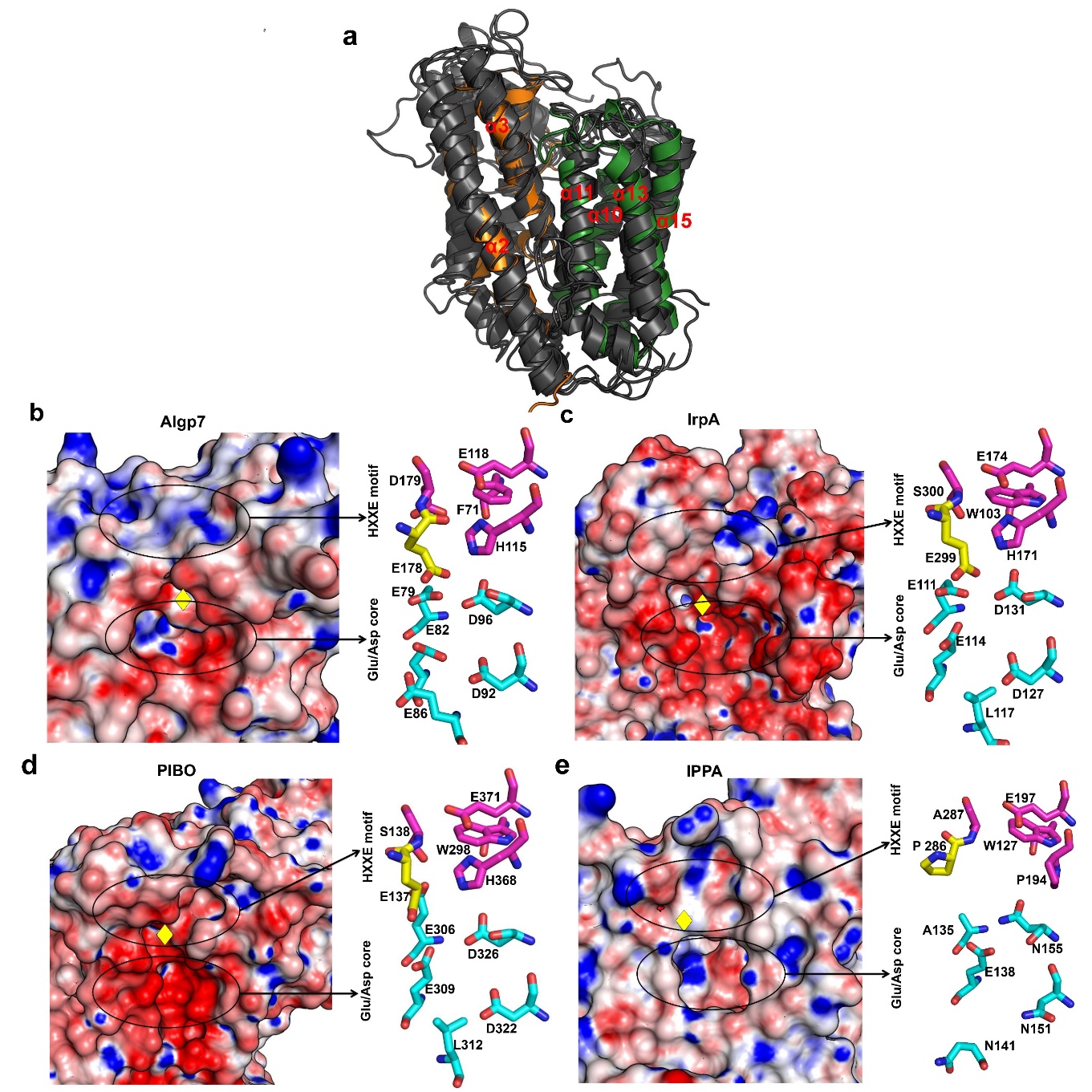
Supplementary Figures**

**Figure S1.** Close-up view of the structural superimpositions, electrostatic surface representations and metal binding pocket comparisons of EfeM with other Algp7/imelysin-like family members. The electrostatic potentials followed by the amino acid ligands related to Site III/Site IV of EfeM with the equivalent sites (HXXE motif and Glu/Asp core) for other Algp7/imelysin-like family members are highlighted. For the purpose of clarity, all structures are positioned according to the histidine/proline of the HXXE motif of EfeM_Psy_. The remaining details are the same as in Fig.1. **a** Structure superimposition for EfeM_Psy_ (orange and dark green cartoon) and the remaining Algp7/imelysin-like family members (dark gray cartoons) using PDBeFold; **b** Algp7 from *Spingomonas* sp A1 (PDB code: 3AT7); **c** IrpA from *Parabacteroides distasonis* ATCC 8503 (PDB code: 4ECG); **d** PIBO from *Bacteroides ovatus* ATCC 8483 (PDB code: 3OYV); **e** IPPA from *Psychrobacter arcticum* 273-4 (PDB code: 3PF0).

**
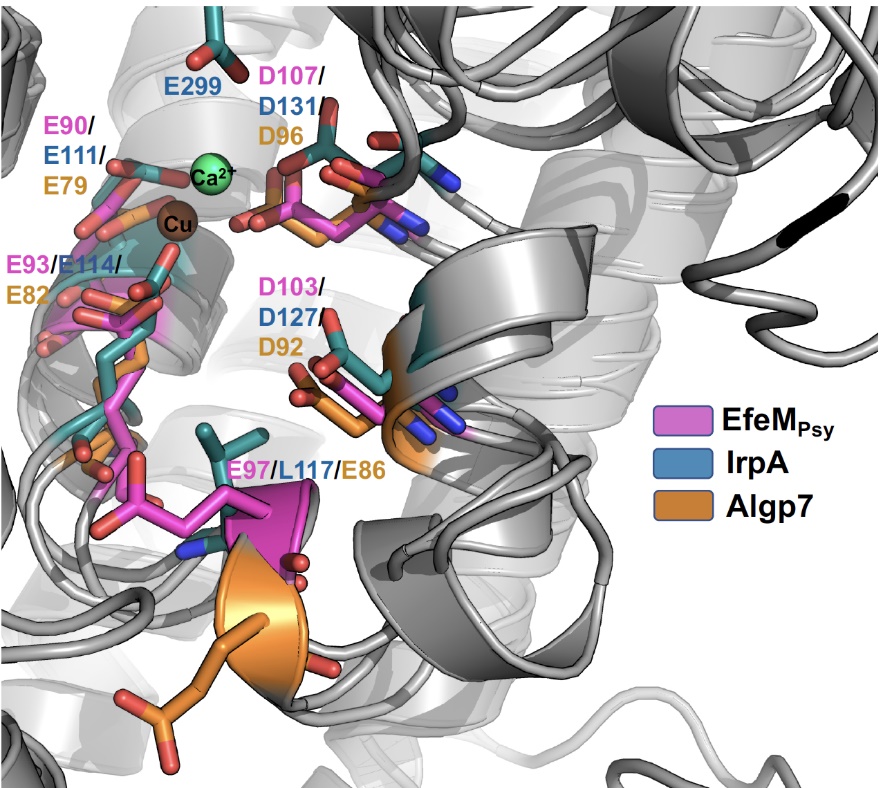
Figure S2**. Comparison of putative Site IV binding pocket of EfeM_Psy_ with metal bound IrpA and Algp7 crystal structures. The structural mapping of metal binding cleft of IrpA and Algp7 revealed promising superimposition of metal binding cleft on to putative Site IV binding pocket of EfeM_Psy_. In addition, the amino acids ligands involved in interacting with calcium and copper for IrpA and Algp7 are also found to Glu/Asp enriched which is concurrent with proposed Glu/Asp enriched residues (Glu90, Glu93, Asp103 and Asp107) of Site IV for EfeM. From the PDBSUM analysis, the calcium ion (at position 504) observed in IrpA are coordinated by residues Glu111, Glu114, Asp131 and Glu299 of IrpA whereas Glu79, Glu82, Asp96 and Gu178 were the key amino acid ligands lining the copper site of Algp7

**Figure S3.** Estimation of the binding affinity and stoichiometric calculations from EfeM – Zn^2+^/Cu^2+^ SRCD titrations. The dissociation constant (K_d_) and stoichiometry were estimated by fitting SRCD data points at 222 nm
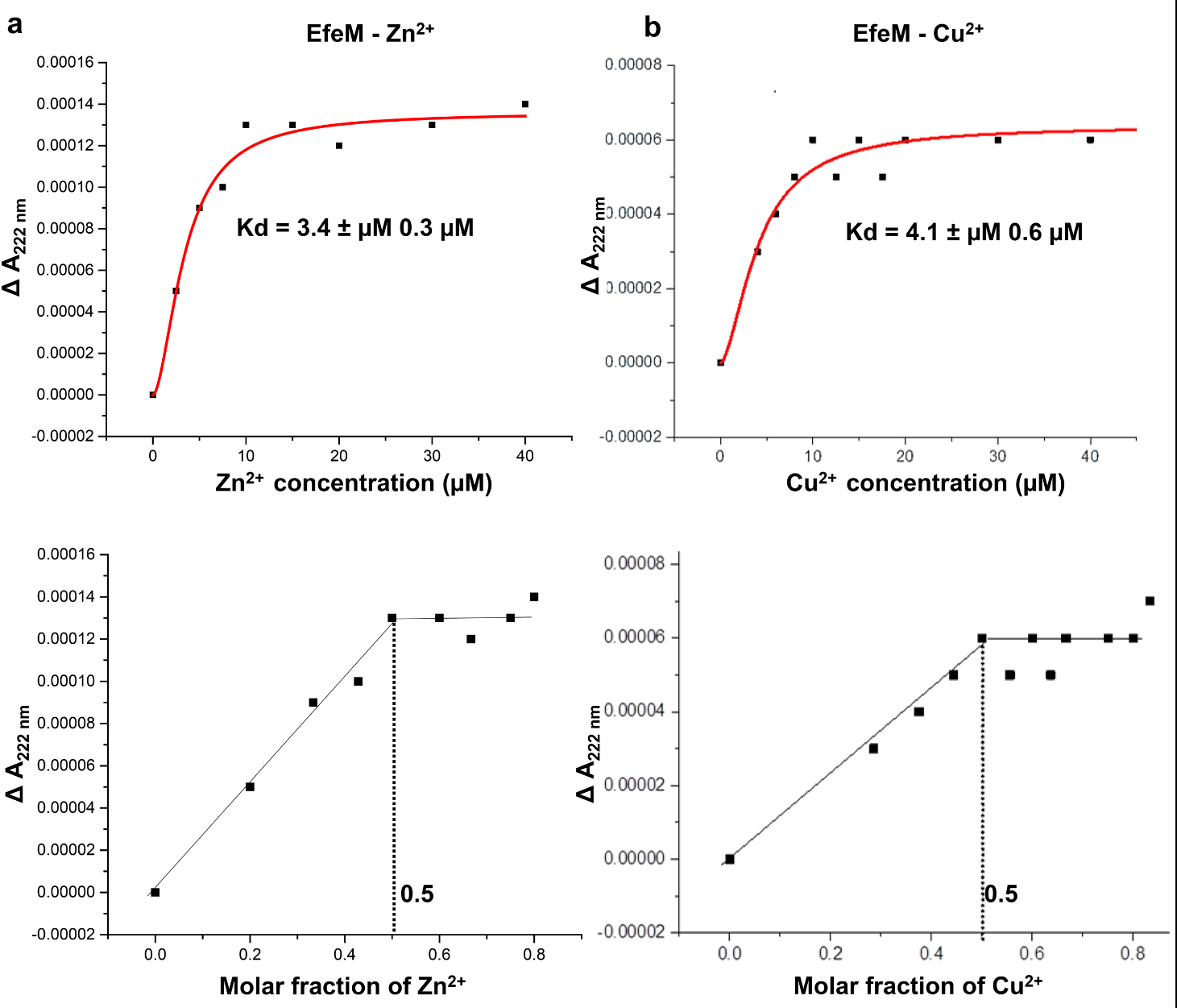
against Zn^2+^ / Cu^2+^ concentration (µM) and molar fractions, respectively, using the CDApps from software, as provided by Diamond B23 Beamline.  **a** Binding profile for EfeM_Psy_ with Zn^2+^ ; **b** Binding profile for EfeM_Psy_ with Cu^2+^. The molar fraction of 0.5 for Zn^2+^ and Cu^2+^ indicates that the complex of EfeM_Psy_ and metals (Zn^2+^ and Cu^2+^) is at a 1:1 ratio.

**
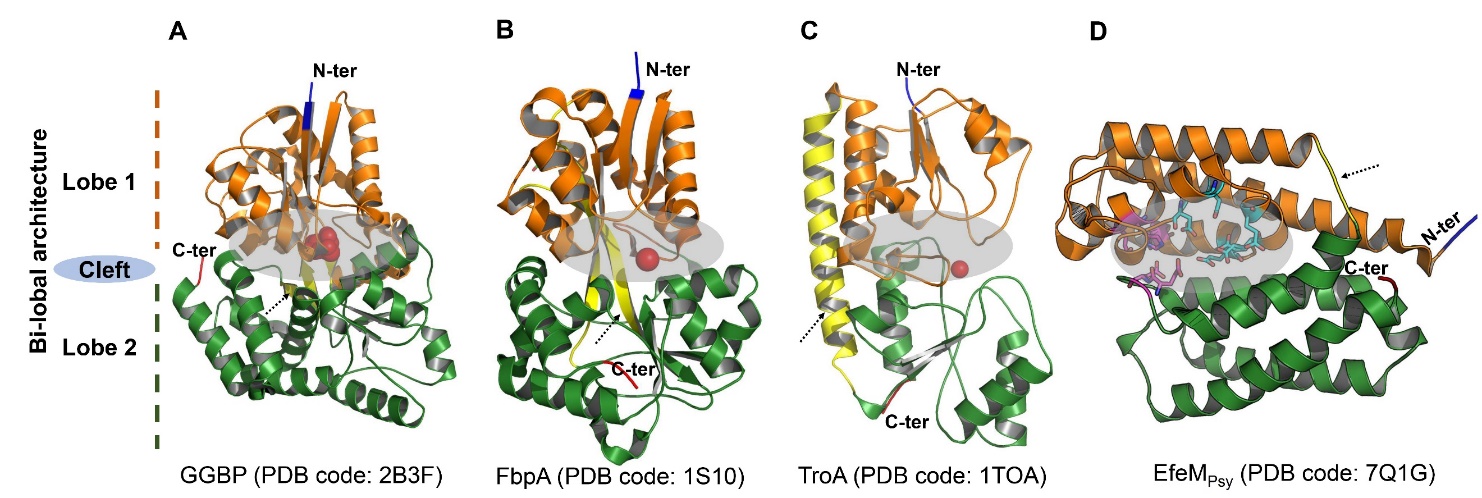
Figure S4.** Structure based comparison of EfeM_Psy_ with other pSBP structures. Overall, EfeM_Psy_ resembles the general SBP fold possessing two globular domains connected by a hinge region with the inter-domain ligand-binding cleft in between. The hinge region (either strands, helix or short loops) are shown in yellow for all structures. The two globular domains with either alpha/beta (GGBP, FbpA and TroA) and alpha only (EfeM_Psy_) in each case are represented as light orange and dark green, respectively. The inter-domain ligand-binding cleft with their substrates/solutes for GGBP, FbpA, TroA are highlighted in semitransparent light gray oval boxes and red spheres, respectively. For EfeM_Psy_, inter-domain ligand-binding cleft is highlighted with potential amino acid ligands lining Site III and Site IV as light magenta and light cyan, respectively. The N- and C-terminal regions for all structures are colored in blue and red, respectively. **a** Cartoon representation of Glucose/Galactose binding protein (GGBP) from *Thermus thermophilus* (Cuneo et al. 2006) (PDB code: 2B3F); **b** Ferric binding protein A (FbpA) from *Mannheimia haemolytica* (Shouldice et al. 2004) (PDB code: 1SI0); **c** Periplasmic zinc binding protein (TroA) from *Treponema pallidum* (Lee et al. 1999) (PDB code: 1TOA) and **d** EfeM_Psy_ structure (PDB code: 7Q1G).


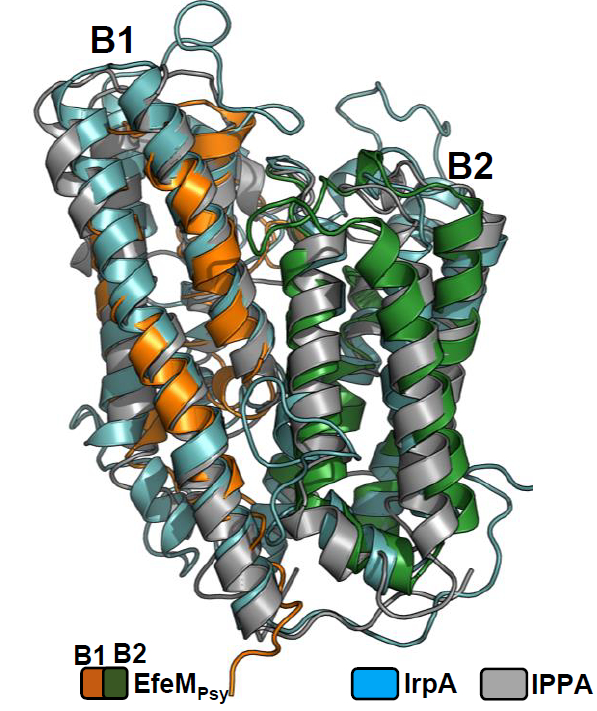
**Figure S5.** Overall structural superimpositions between EfeM and other representative Algp7/imelysin-like family members; IrpA (PDB code:4ECG) and IPPA (PDB code:3PF0). The remaining details for EfeM_Psy_ are same as in Fig.1. The overall M75 domain with two four-helix bundles (B1 and B2) are conserved between all these proteins.


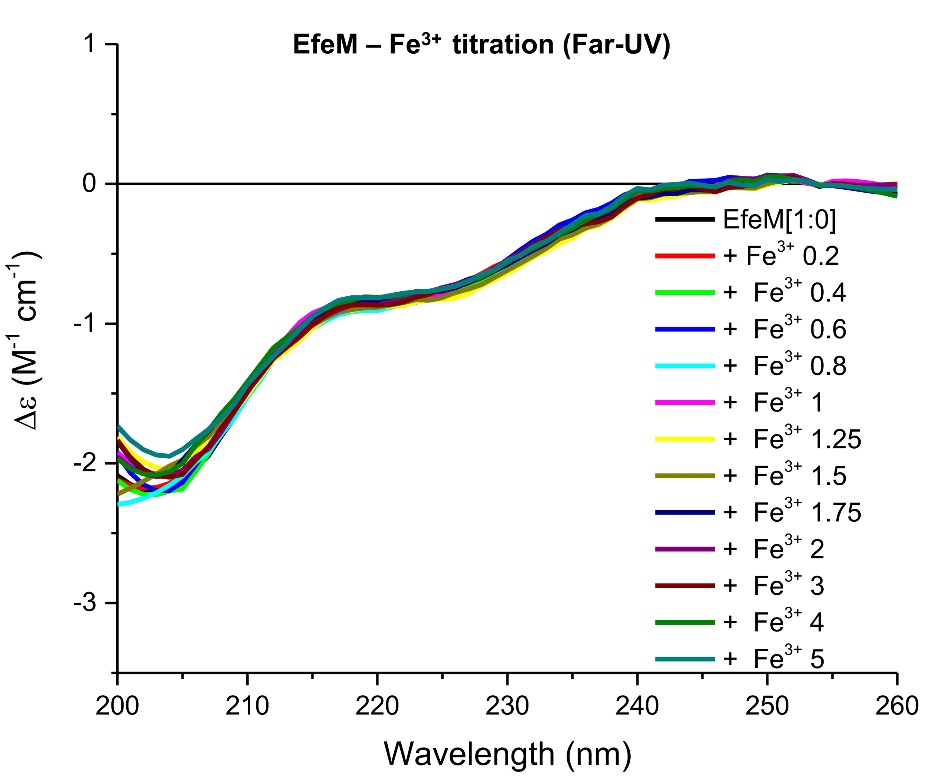
**Figure S6.** Far–UV (200-260 nm) SRCD spectra of apo EfeM_Psy_ and its titration with Fe^3+^. SRCD spectra of EfeM_Psy_ with Fe^3+^ additions with molar stoichiometry starting from 0.2 (2 μM) to 5 equivalents (50 μM). No significant conformational changes observed after additions. The other details are as in Fig. 4.

**Table S1: List of representative members from EfeO/EfeM and Algp7-imelysin like family**

| Entry name | Organism | UNIPROTKB accession code |
| --- | --- | --- |
| EfeM_Psy | EfeM_*Pseudomonas_syringae* | Q4ZR20 |
| EfeM_Rhopb | EfeM_*Rhodopseudomonas_palustris* | Q217C2 |
| EfeM_Psef5 | EfeM_*Pseudomonas_fluorescens* | Q4KBM0 |
| EfeM_Chrsd | EfeM_*Chromohalobacter_salexigens* | Q1QSA3 |
| EfeM_Pardp | EfeM_*Paracoccus_denitrificans* | A1B2T9 |
| EfeO_Ecoli | EfeO_*Escherichia_coli* | P0AB24 |
| EfeO_Shifl | EfeO_*Shigella_flexneri* | P0AB25 |
| EfeO_Yere8 | EfeO_*Yersinia_enterocolitica* | A1JMT4 |
| EfeO_Shiss | EfeO_*Shigella_sonnei* | Q3Z397 |
| EfeO_Serp5 | EfeO_*Serratia_proteamaculans* | A8GFY9 |
| Algp7_Sping | Algp7_*Spingomonas* | Q25C86 |

References

Cuneo MJ et al. (2006) The crystal structure of a thermophilic glucose binding protein reveals adaptations that interconvert mono and di-saccharide binding sites. J Mol Biol 362:259-70. doi: 10.1016/j.jmb.2006.06.084.

Shouldice SR et al. (2004) Structural basis for iron binding and release by a novel class of periplasmic

iron-binding proteins found in gram-negative pathogens. J Bacteriol 186:3903-10. doi: 10.1128/JB.186.12.3903-3910.2004.

Lee et al. (1999) Treponema pallidum TroA is a periplasmic zinc-binding protein with a helical backbone. Nat Struct Biol 6:628-33. doi: 10.1038/10677.

1. Present address: Sussex Drug Discovery Centre, School of Life Sciences, University of Sussex, Falmer, Brighton, BN19QG, UK [↑](#footnote-ref-2)
